# Delayed PARP-1 Inhibition Alleviates Post-stroke Inflammation in Male Versus Female Mice: Differences and Similarities

**DOI:** 10.3389/fncel.2020.00077

**Published:** 2020-04-03

**Authors:** Jian Chen, Xiaoxi Li, Siyi Xu, Meijuan Zhang, Zhengzheng Wu, Xi Zhang, Yun Xu, Yanting Chen

**Affiliations:** ^1^The State Key Laboratory of Pharmaceutical Biotechnology, Department of Neurology, Drum Tower Hospital, Medical School of Nanjing University, Nanjing, China; ^2^Jiangsu Key Laboratory for Molecular Medicine, Medical School of Nanjing University, Nanjing, China; ^3^Jiangsu Province Stroke Center for Diagnosis and Therapy, Nanjing, China

**Keywords:** gender, inflammation, ischemic stroke, PARP-1, microglia

## Abstract

Post-stroke inflammation is almost involved in the whole process of stroke pathogenesis, which serves as a prime target for developing new stroke therapies. Despite known sex differences in the incidence and outcome of stroke, few preclinical or clinical studies take into account sex bias in treatment. Recent evidence suggests that poly (ADP-ribose) polymerase (PARP)-1 inhibitor exerts sex-specific neuroprotection in the ischemic stroke. This study was aimed to investigate the effects of delayed PARP-1 inhibition on post-stroke inflammation and possible sexual dimorphism, and explore the possible relevant mediators. In male and female C57BL/6 mice subjected to transit middle cerebral artery occlusion (MCAO), we found that delayed treatment of PARP-1 inhibitor at 48 h following reperfusion could comparably alleviate neuro-inflammation at 72 h after stroke. Whereas, more remarkable reduction of iNOS and MMP9 induced by PARP-1 inhibition were found in male MCAO mice, and the improvement of behavioral outcomes was more prominent in male MCAO mice. In addition, we further identified that PARP-1 inhibitor might equivalently suppress microglial activation in males and females *in vivo* and *in vitro.* With proteomic analysis and western blotting assay, it was found that stroke-induced peroxiredoxin-1 (Prx1) expression was significantly affected by PARP-1 inhibition. Interestingly, injection of recombinant Prx1 into the ischemic core could block the anti-inflammatory effects of PARP-1 inhibitor in the experimental stroke. These findings suggest that PARP-1 inhibitor has effects on regulating microglial activation and post-stroke inflammation in males and females, and holds promise as a novel therapeutic agent for stroke with extended therapeutic time window. Efforts need to be made to delineate the actions of PARP-1 inhibition in stroke, and here we propose that Prx1 might be a critical mediator.

## Introduction

Ischemic stroke is one of the major causes of morbidity and mortality worldwide. To date, intravenous thrombolysis, and thrombectomy are approved efficient therapies for ischemic stroke patients (Lambertsen et al., 2019; Tao et al., 2019; Yang et al., 2019). However, due to the narrow therapeutic time window and a cascade of pathophysiological events induced by reperfusion, the recanalization therapies are only available for a small fraction of stroke patients (Bosetti et al., 2017; Ma et al., 2019). Therefore, novel therapeutic options for cerebral ischemia are urgently needed. New treatments targeting key pathogenic mechanisms are currently being explored in the preclinical and clinical stroke research, either alone or in combination with reperfusion therapies (George and Steinberg, 2015; Bosetti et al., 2017). Inflammation is integral to the whole process of stroke pathogenesis, thus serving as a prime target for the development of new stroke therapies (Shao et al., 2019; Zhu et al., 2019), and potentially providing a clinically accessible time window for initiating therapy (Chen et al., 2014).

Poly (ADP-ribose) polymerase (PARP)-1 is an abundant nuclear protein, which plays a critical role in various DNA repair pathways and in the maintenance of genomic stability (Wang et al., 2016; Ray Chaudhuri and Nussenzweig, 2017). Accumulating evidence reveals that excessive activation of PARP-1 contribute to pathogenesis of many diseases including ischemic stroke (Bai and Canto, 2012; Charriaut-Marlangue et al., 2018). Several PARP inhibitors have entered clinical trials for cancer and other diseases (Curtin and Szabo, 2013; Wang et al., 2016). PARP-1 inhibitor also displays exciting therapeutic potential in experimental and clinical stroke studies. PJ34, a PARP inhibitor, can inhibit the activation from PARP to PAR and preserve intracellular NAD + levels, thus improves mitochondrial function (Zha et al., 2018). Xu et al. (2016) suggested that PJ34 (given immediately after ischemia followed by one injection per day until the third day) along could block microglial activation and alleviate post-stroke neuro-inflammation. Besides, PJ34 (given combined with rt-PA at 6 h after ischemia) was also proven to reduce rt-PA-induced BBB breakage and hemorrhagic transformations (Haddad et al., 2013; Teng et al., 2013). Moreover, the recent clinical availability of the PARP inhibitor Lynparza (olaparib) has been proposed for potential therapeutic repurposing for non-oncological indications including stroke (Berger et al., 2018).

Sexual dimorphism was well-documented in the pathophysiology, incidence and severity of stroke. Importantly, post-stroke inflammation was sex-specific, for instance, inflammatory cells like resident microglia and peripheral T cells, and response to CNS injury with sextual difference (Ahnstedt and McCullough, 2019; Kerr et al., 2019). However, few investigations take into account possible gender bias of stroke patients in the response to treatments in the preclinical research (Sohrabji et al., 2017), thereby potential gender associated pathogenesis could be neglected. Recent evidence indicates that PARP activation plays a key role in the ischemic sexual dimorphism (Yuan et al., 2009). PARP-1 stimulates the production of poly (ADP-ribose) polymerase (PAR) and release of apoptosis inducing factor (AIF) from the mitochondria in males, leading to cell death, whereas the PARP-1/AIF pathway does not play a causal role in stroke-induced cell death in females, which might be associated the finding that immediately PARP-1 inhibition after ischemia alleviate ischemic brain injury only in males (Lang and McCullough, 2008; Yuan et al., 2009). Interestingly, several previous studies have shown that PARP inhibition preferentially protects males in the experimental stroke (Hagberg et al., 2004; Waye, 2009; Yuan et al., 2009). Moreover, in an open-label evaluator-blinded clinical study, gender-dependent effects were observed in the neuroprotection of oral minocycline (a putative PARP inhibitor) treatment in the ischemic stroke (Amiri-Nikpour et al., 2015). Recognition of the possible sex differences in the stroke research will play an critical role in the translational success of novel therapeutic agents.

In this study, we attempted to investigate the effects of delayed PARP-1 inhibition on post-stroke neuro-inflammation and behavioral performance in male and female mice. We further aimed to identify key mediators underlying the actions of PARP-1 inhibitor in the ischemic stroke. Moreover, PARP-1 inhibitor was administrated at 48 h following ischemia/referfusion, in order to comfirm that whether PARP-1 inhibitor could potentially provide an extended time window for stroke treatment.

## Materials and Methods

### Animals

Adult male and female C57BL/6 mice (22–26 g, 8 to 10 weeks old) were purchased from the Model Animal Research Center of Nanjing University (Nanjing, China). All animals were housed in a specific pathogen-free facility with a 12 h light/dark cycle. Food and water were available *ad libitum*. All efforts were made to minimize animal suffering and the number of animals used. All animal experiments were approved by the Institutional Animal Care and Use Committee of Nanjing University.

### Middle Cerebral Artery Occlusion

The transient middle cerebral artery occlusion (MCAO) was conducted to induce focal ischemia as previously described (Chen et al., 2019). Briefly, mice were anesthetized with 1.5% isoflurane in a 68.5% N_2_O/30% O_2_ mixture. Transient focal ischemia was induced by intraluminal occlusion of the right middle cerebral artery (MCA) for 50 min. At first, a 1-cm long midline neck incision was made and the right external carotid artery (ECA) was exposed and isolated from the adjacent tissue. Then, a silicone-rubber-coated 6-0 nylon filament (Doccol, United States) was inserted into the internal carotid artery via the proximal ECA and further inserted to the circle of Willis to achieve the MCAO. After 50 min of occlusion, the monofilament was withdrawn to allow reperfusion. Sham-operated mice were subjected to the same procedure without occlusion of MCA. Mice exposed to MCAO were placed in a 35°C nursing box for anesthesia recovery.

### PARP-1 Inhibitor PJ34 and Recombinant Mouse Peroxiredoxin 1 (Prx1) Injection

The MCAO mice were intraperitoneally (i.p.) administrated with either vehicle or N-(6-Oxo-5.6-dihydrophenanthridin-2-yl)- (N, N-dimethylamino) acetamide hydrochloride (PJ34, 30 mg/kg, Tocris Bioscience, United States) 48 h after MCAO. For Prx1 injection, 48 h after MCAO, mice were fixed in a stereotactic frame, and Prx1 (Sigma, United States, 2 μl/mouse) was injected to the right ischemic striatum using the following microinjection coordinates: anteroposterior, −0.5 mm; lateral, 2.0 mm; and ventral, 3.5 mm.

### Quantitative Real-Time Polymerase Chain Reaction

Total RNA was extracted from mouse brain tissue (mainly include cortex and striatum) and cultures using Trizol reagent (Invitrogen, United States). The cDNA was synthesized using a Prime Script RT reagent kit (Takara, United States) according to the protocol of the manufacturer. Real-time PCR was performed using SYBR Green (Takara, United States) with the ABI 7500 PCR instrument (Applied Biosystems, United States) as previously described (Liu et al., 2019). The primer sequences used in this study were listed as follows:

GAPDH forward primer, 5′-GCCAAGGCTGTGGGCAAGGT-3′’;

GAPDH reverse primer, 5′’-TCTCCAGGCGGCACGTCAGA-3′’;

INOS forward primer, 5′’-CAAGCACCTTGGAAGAGGAG-3′’;

INOS reverse primer, 5′’-AAGGCCAAACACAGCATACC-3′’;

IL-1β forward primer, 5′’-GCAACTGTTCCTGAACTCAACT-3′’;

IL-1β reverse primer, 5′’-ATCTTTTGGGGTCCGTCAACT-3′’;

TNFα forward primer, 5′’-CCCTCACACTCAGATCATCTTCT-3′’;

TNFα reverse primer, 5′’-GCTACGACGTGGGCTACAG -3′’;

MMP9 forward primer, 5′’-CTGGACAGCCAGACACTAAAG-3′’;

MMP9 reverse primer, 5′’-CTCGCGGCAAGTCTTCAGAG-3′’;

CD11b forward primer, 5′’-CCAAGACGATCTCAGCATCA-3′’;

CD11b reverse primer, 5′’-TTCTGGCTTGCTGAATCCTT-3′’;

Iba-1 forward primer, 5′’-TCCTCCGGCCCATGATTAAAG-3′’;

Iba-1 reverse primer, 5′’-CTGTCTGGCTGCCCATTCT-3′’;

GFAP forward primer, 5′’-CGGAGACGCATCACCTCTG-3′’;

GFAP reverse primer, 5′’-AGGGAGTGGAGGAGTCATTCG-3′’;

CD16 forward primer, 5′’-TTTGGACACCCAGATGTTTCAG-3′’;

CD16 reverse primer, 5′’-GTCTTCCTTGAGCACCTGGATC-3′’;

CD206 forward primer, 5′’-CAAGGAAGGTTGGCATTTGT-3′’;

CD206 reverse primer, 5′’-CCTTTCAGTCCTTTGCAAGC-3′’;

TGF-β forward primer, 5′’-AACTATTGCTTCAGCTCCACAGAG-3′’;

TGF-β reverse primer, 5′’-AGTTGGATGGTAGCCCTTG-3′’;

Prx1 forward primer, 5′’- CAA GTG ATT GGC GCT-3′’;

Prx1 reverse primer, 5′’-GGT CCC AAT CCT CCT TG-3′’;

Prx6 forward primer, 5′’-CCAGCACTGATCTAGGTCTCC-3′’;

Prx6 reverse primer, 5′’-ATGCCCCATGAATCTCCCAG-3′’.

### Western Blotting

Tissues were resected from the brain of MCAO mice and further homogenized and lysed using the lysis buffer (Thermo Fisher Scientific, United States) containing 1% protease inhibitor cocktail (Sigma-Aldrich, United States) as described previously (Deng et al., 2019). Protein concentration was determined using Bicinchoninic Acid assay (Beyotime Biotechnology, China). Equal amounts of total protein from each sample were separated by SDS-PAGE and blotted onto PVDF membranes. Then, the membranes were incubated with primary antibodies, including CD11b (1:1000; Abcam, United States), GFAP (1:1000; Abcam, United States), Prx1 (1:1000; Abcam, United States), Prx6 (1:1000; Abcam, United States), and β-Actin (1:5000; Bio-Rad, United States). Secondary antibodies were horseradish peroxidase (HRP)–conjugated antibodies (Bio-Rad, United States). Protein bands were detected using a chemiluminescent substrate kit (Millipore, United States). Image-Pro Plus (National Institutes of Health, United States) was used to analyze the intensities of the bands.

### Behavior Test

The modified neurological severity score (mNSS) and grip strength were employed to assess the neurological performance of the MCAO mice 24 h after PJ34 administration as described previously (Meng et al., 2019). mNSS score: the test was performed mainly to assess the motor, reflex, sensory, and balance deficits with a score range from 0 to 18 (0: normal; 18: maximal deficit score). Grip strength: Grip strength was measured using a Panlab machine (LE902, United States). Briefly, the mouse was allowed to hold the platform of the grip-strength machine using the front paws and then the mouse was pulled in a straight line away from the platform, each mouse was tested for three times and maximum grip strength was recorded. Neurobehavioral performance was evaluated by an observer blinded to the experiments.

### Primary Microglial Culture and Drug Treatment

Primary microglia were isolated and purified from brains from postnatal 1–2 days C57BL/6 mice, with sex determined via identifying sex chromosomes as described previously (Posynick and Brown, 2019). The cerebral cortex tissues of mice were dissected and digested in Tryple (Thermo Fisher Scientific, United States) for 5 min at 37°C. After centrifugation, the cell mixture was suspended and passed through a 70 μM pore filter. Finally, cells were planted in 75 cm^2^ flasks in 5% CO2 at 37°C, and the medium was half-changed at day 5. After culturing for 2 weeks, microglial cells were obtained by gently shaking the flasks. The microglia were collected, centrifuged, and then replated onto 12-well plates at a density of 2 × 10^5^/well.

For drug administration, PARP-1 inhibitor DPQ (25 μM) was applied to the microglial culture medium combined with 500 ng/μl lipopolysaccharide (LPS) (Sigma-Aldrich, United States). After 24 h, cells were harvested for the following experiments.

### Proteomic and Microarray Processing Analysis

Proteomics analysis was performed by AB SCIEX TripleTOF 5600 mass spectrometer (AB SCIEX, United States) equipped with a liquid chromatography-tandem mass spectrometry (LC-MS/MS) system. Proteins (100 μg) of each sample were resolved on SDS polyacry-lamide gels and then stained with Coomassie Blue G-250 for 4 h. After distaining in ultrapure H_2_O, the gel was cut into blocks, and digested using trypsin. After being cleaned, desalted, and vacuum-dried, the peptides were analyzed. The Agilent Sure Print G3 Mouse GE V2.0 Microarray plates were conducted by Shanghai oebiotech Company of China. Briefly, total RNA was acquired from mouse brain tissue and quantified by the NanoDrop ND-2000 (Thermo Fisher Scientific, United States). Then, RNA was purified and transcribed to double strand cDNA. Then, the cDNA was synthesized into cRNA to be hybridized onto the microarray and scanned by Agilent Scanner G2505C (Agilent Technologies, China) to acquire images and analyzed using Feature Extraction software (version10.7.1.1, Agilent Technologies, China). The bioinformatics analysis of differentially expression proteins and genes were conducted by GO (Gene Ontology) and KEGG (Kyoto Encyclopedia of Genes and Genomes) analysis.

### Nitric Oxide Measurements

Production of nitric oxide (NO) *in vitro* was determined as described previously (Zhang et al., 2018). Briefly, after co-treatment of DPQ (25 μM) and LPS (500 ng/μl) for 24 h, the supernatant was mixed with an equal volume of Griess reagent and then incubated at room temperature for 15 min. Absorbance at 540 nm was measured to determine NO production. Four replicate wells were used in this experiment.

### Statistical Analysis

Comparisons between groups were carried out by one-way analysis of variance (ANOVA) followed by Bonferroni-corrected *post hoc* tests. Comparative differences were considered significant at *P* < 0.05. The statistical software package Sigma Stat 11.5 (SPSS) was used to perform all analysis. Data are presented as means ± SEM.

## Results

### PARP-1 Inhibitor PJ34 Alleviated Post-stroke Neuro-Inflammation and Neurological Deficits in Males Versus Females

**FIGURE 1:**
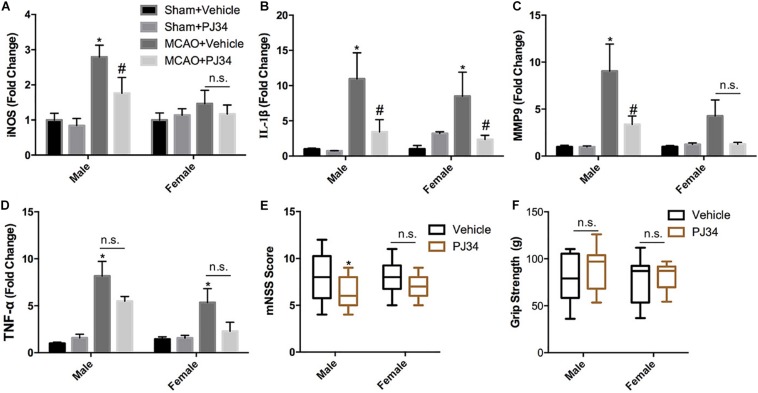
PARP-1 inhibitor PJ34 alleviated post-stroke neuro-inflammation and neurological deficits in males versus females. PARP-1 inhibitor PJ34 was administrated to both males and females at 48 h after MCAO, 24 h later, ipsilateral cortexes were isolated and real-time PCR was performed to detect the mRNA level of pro-inflammatory cytokines, including **(A)** iNOS, **(B)** IL-1β, **(C)** MMP9, and **(D)** TNF-α. Values are presented as mean ± SEM for three mice in each group. *P < 0.05 versus sham + Vehicle groups, #*P* < 0.05 versus MCAO + Vehicle groups. The behavioral performance of male and female MCAO mice was evaluated by **(E)** mNSS and **(F)** Grip strength. Values are the median (25th and 75th percentiles), *n* = 14–16. *P < 0.05 versus Vehicle groups. n.s., no significance.

This study was commenced by investigating whether delayed PARP-1 inhibition could mitigate post-stroke inflammation and neurological deficits, and whether there was a sex bias in the effects of PARP-1 inhibition. PARP-1 inhibitor PJ34 was given to both male and female MCAO mice after 48 h of reperfusion, 24 h following PJ34 administration, inflammatory profiles in all groups of mice were assessed. Quantitative real-time PCR was employed to detect inflammatory cytokines in the ipsilateral cortex of the MCAO mice. As shown in Figure 1A, cerebral ischemia induced substantial inducible nitric oxide synthase (iNOS) expression in males, which was significantly attenuated by PJ34 treatment. Whereas, no evident induction of iNOS was observed in female MCAO mice. In addition, we found that cerebral ischemia resulted in remarkably increased mRNA expression of interleukin (IL)-1β in both male and female mice, which were consistently reversed by PRAP-1 inhibition (Figure 1B). Alteration of matrix metallopeptidase 9 (MMP9) displayed a similar pattern as iNOS (Figure 1C). Tumor necrosis factor (TNF) -α was significantly increased in males and females after ischemia. A downward trend of TNF -α by PARP-1 inhibition was observed in both genders (Figure 1D). Behavioral tests were further performed to evaluate the therapeutic effects of delayed PARP-1 inhibition on neurological deficit of MCAO mice. As shown in Figure 1E, delayed administration of PJ34 improved neurological functions in the male group with a lower mNSS, whereas a minor improvement of the neurological deficits was found in the female group (Figure 1E). No significant improvement of grip strength was observed in both male and female MCAO mice (Figure 1F). These data demonstrated that delayed PARP-1 inhibitor PJ34 administration could mitigate post-stroke neuro-inflammation in both males and females. This regulatory effects of delayed PARP-1 inhibition, however, were not completely consistent in both genders, and the improvement of neurological deficits was more pronounced in male MCAO mice.

### PARP-1 Regulates Inflammatory Response After Stroke in Males Versus Females

**FIGURE 2:**
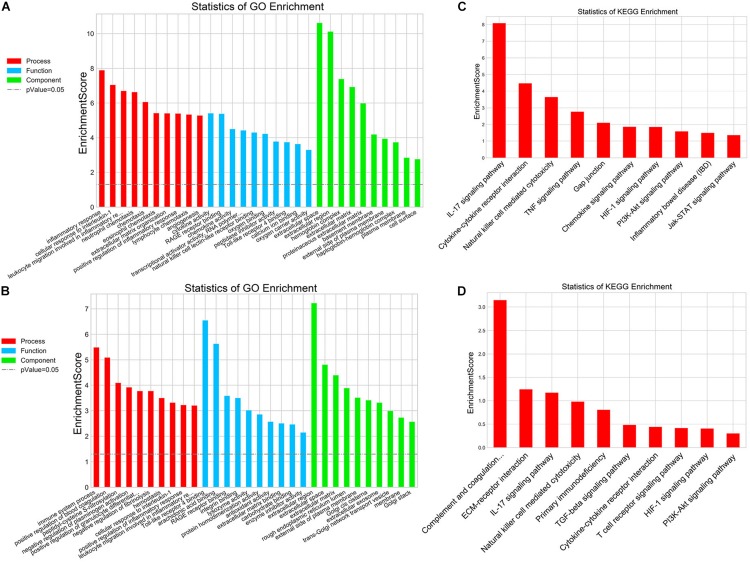
PARP-1 regulated inflammatory response after stroke in males and females. GO analysis of the differentially expressed genes (MCAO + Vehicle versus MCAO + PJ34, fold change ≥ 2, *p* < 0.05) in **(A)** the male group and **(B)** the female group, including three aspects: biological process, cellular component, and molecular function. KEGG pathway analysis of the differentially expressed genes (MCAO + Vehicle versus MCAO + PJ34, fold change ≥ 2, *p* < 0.05) in **(C)** the male group and **(D)** the female group, *n* = 3 mice per group.

In addition, mRNA microarray was further performed to explore the influence of delayed administration of PJ34 on the molecular profile in the ischemic cortex in both males and females (*n* = 3 mice per group). GO analysis was employed to evaluate the biological characteristics of the differentially expressed genes (fold change ≥ 2.0 and *p* < 0.05) in three aspects: biological process, molecular function and cellular component. As shown in Figures 2A,B, GO analysis indicated that differentially expressed genes affected by delayed PJ34 treatment after MCAO were mainly associated with inflammatory response regulation in both males and females. Briefly, in the male group, differentially expressed genes were mainly enriched in inflammatory response, cellular response to IL-1 and leukocyte migration involved in inflammatory response, et cetera. Whereas, in the female group, differentially expressed genes were mostly related to immune system process, positive regulation of blood coagulation and peptidyl- cysteine S- nitrosylation. Moreover, KEGG pathway analysis was performed to analyze the potential pathways altered by PJ34 in males and females in stroke. Consistent with the results of GO analysis, the differentially expressed genes affected by PJ34 were abundantly enriched in pathways that are implicated in modulating inflammation. In the male MCAO mice, the differentially expressed genes were mainly located in IL-17 signaling pathway, cytokine-cytokine receptor interaction and Natural killer cell mediated cytotoxicity, et cetera (Figure 2C). In the female group, complement and coagulation cascades, ECM-receptor interaction, and IL-17 signaling pathways were significantly influenced by delayed treatment of PARP-1 inhibitor in the ischemic stroke (Figure 2D). These markedly affected biological processes and pathways might account for the similarities and differences underlying the effects of PARP-1 inhibition in post-stroke inflammation.

### PARP-1 Inhibition Modulates Microglial Activation After Cerebral Ischemia

**FIGURE 3:**
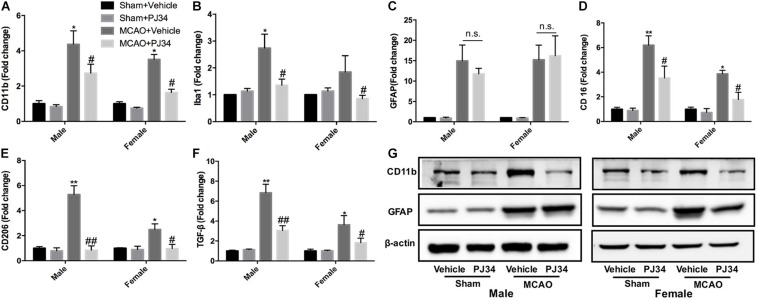
PARP-1 inhibition modulated microglial activation after cerebral ischemia. Relative mRNA level of inflammatory mediators in cortexes from male and female groups were detected via real-time PCR, including **(A)** CD11b, **(B)** Iba-1, **(C)** GFAP, **(D)** CD16, **(E)** CD206, and **(F)** TGF-β. Values are presented as mean ± SEM for three mice in each group. *P < 0.05 and **P < 0.01 versus sham + Vehicle groups, #*P* < 0.05 and ##*P* < 0.01 versus MCAO + Vehicle groups. **(G)** CD11b and GFAP protein level were detected via western blotting. n.s., no significance.

Microglia and astrocytes are the two main resident components of resident immune cells in the ischemic brain (Cserep et al., 2019). Since delayed treatment of PJ34 was found to suppress neuro-inflammation in the ischemic brain, we further explored whether delayed PARP-1 inhibition could block the activation of microglia or astrocytes. Microglial and astrocytic activation were evaluated as described previously (Cheon et al., 2017; Jiang et al., 2017). CD11b and Iba-1 were used as microglial activation markers, and GFAP was used to assess the activation level of astrocytes. At first, we evaluated the mRNA expression of CD11b, Iba-1, and GFAP via quantitative real-time PCR. It was demonstrated that delayed PJ34 administration significantly reduced the mRNA level of CD11b in the ischemic brain in both of the male and female groups (Figure 3A). In addition, Iba-1 expression was also down-regulated by PARP-1 inhibition in both males and females (Figure 3B). On the contrary, delayed PARP-1 suppression did not affect GFAP mRNA level (Figure 3C). To further examine whether PJ34 affected the polarization of microglia in the ischemic brain, pro-inflammatory microglial marker (CD16) and anti-inflammatory microglial markers (CD206 and TGF-β) were detected using real-time PCR, our findings indicated that PJ34 reduced expression of both CD16, CD206, and TGF-β in the MCAO mice of both genders (Figures 3D–F), which suggested that PARP-1 inhibitor simultaneously affected the pro-inflammatory and anti-inflammatory microglial activation induced by cerebral ischemia. Furthermore, western blotting assay was further performed to validate the protein levels of inflammatory markers. In consistent with the alteration of mRNA expression, PJ34 down-regulated the protein level of CD11b in both of the male and female MCAO mice, while GFAP protein level was not affected (Figure 3G and Supplementary Figure S1). Thus, our results indicated that PARP-1 inhibitor might attenuate neuro-inflammation by targeting the microglial activation after stroke.

### PARP-1 Inhibition Alleviates Microglial Activation *in vitro*

**FIGURE 4:**
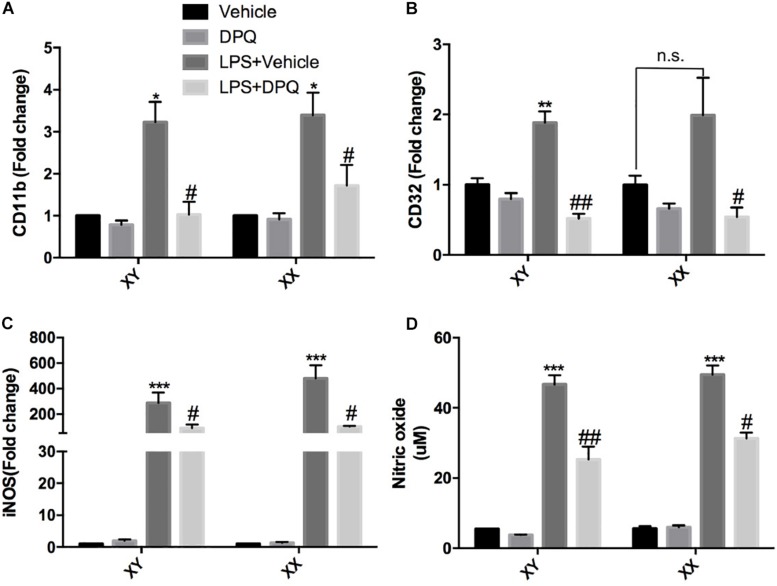
PARP-1 inhibition alleviated microglial activation *in vitro.* Relative mRNA level of inflammatory factors in sex-segregated (XY, male; XX, female) primary microglia were detected via real-time PCR, including **(A)** CD11b, **(B)** CD32, and **(C)** iNOS. **(D)** NO production measured by Griess assay. Values are presented as mean ± SEM in each group of three to four independent experimental procedures. *P < 0.05, **P < 0.01, and ***P < 0.001 versus Vehicle groups, #*P* < 0.05 and ##*P* < 0.01 versus LPS + Vehicle groups. n.s., no significance.

To further validate that PARP-1 inhibition block microglial activation *in vitro*, primary microglia with sex identified were cultured and subjected to co-treatment of LPS and PARP-1 inhibitor DPQ. Real-time PCR results demonstrated that PARP-1 inhibitor DPQ blocked microglial activation induced by LPS, with decreased mRNA levels of CD11b and CD32 in both of the male and female groups (Figures 4A,B). In addition, PARP-1 inhibitor could also mitigate the LPS- induced elevation of iNOS mRNA levels and NO release (Figures 4C,D).

### PARP-1 Suppression Affects Peroxiredoxins in MCAO Mice

**FIGURE 5:**
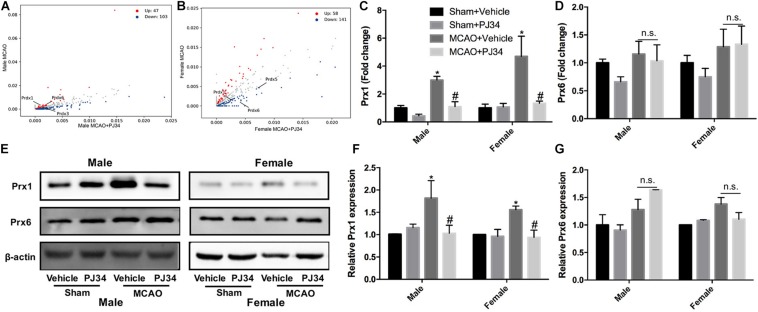
PARP-1 suppression regulated peroxiredoxins in MCAO mice. Scatter plots assessing the variations in protein expression between vehicle and PJ34 treatment group in **(A)** males and **(B)** females. Relative expression of **(C)** Prx1 and **(D)** Prx6 mRNA level were detected via quantitative PCR. Values are presented as mean ± SEM in each group of three independent experimental procedures. *P < 0.05 versus sham + Vehicle groups, #*P* < 0.05 versus MCAO + Vehicle groups. **(E)** Representative western blotting results of Prx1 and Prx6 in both male and female MCAO mice. **(F)** Quantification of the western blotting result of Prx1. **(G)** Quantification of the western blotting result of Prx6. Values are presented as mean ± SEM for three mice in each group. *P < 0.05 versus sham + Vehicle groups, #*P* < 0.05 versus MCAO + Vehicle groups. n.s., no significance.

To elucidate the molecular mechanisms underlying the effects of PARP-1 inhibition in stroke, protein mass spectrometry was conducted to screen key mediators. Peroxiredoxins (Prxs) are a family of thioredoxin peroxidases that activate the Toll-like receptor 4 in immune cells and initiate neuro-inflammation after ischemic stroke (Garcia-Bonilla and Iadecola, 2012; Liu et al., 2018). As shown in Figures 5A,B, Prxs were significantly altered by PJ34 both in male and female MCAO mice. Real-time PCR was used to detect the mRNA levels of peroxiredoxin 1 (Prx1) and peroxiredoxin 6 (Prx6),which are abundant in microglia. A substantial decrease of Prx1 was observed in both male and female MCAO mice (Figure 5C), whereas no evident alteration of Prx6 mRNA level was found in MCAO mice of both genders (Figure 5D). Consistently, Prx1 protein level in the ischemic cortex from either male or female mice was significantly reduced by PJ34, while Prx6 protein level was not affected (Figures 5E–G).

### Exogenous Prx1 Reverses the Effects of PARP-1 Inhibition in the Experimental Stroke

**FIGURE 6:**
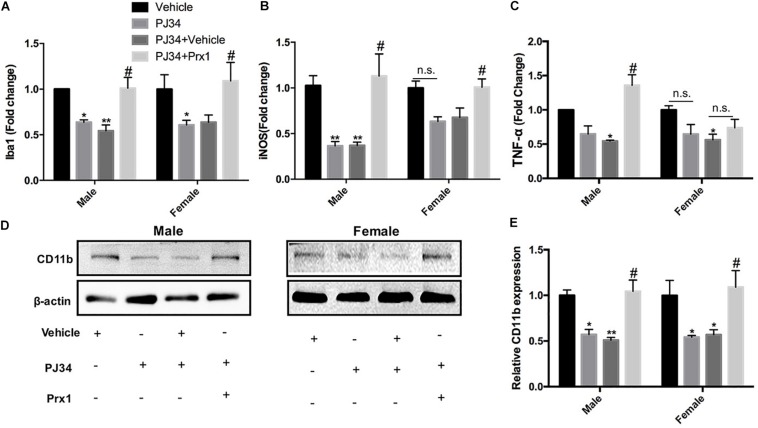
Exogenous recombinant Prx1 reversed the effects of PARP-1 inhibition in the experimental stroke. Relative mRNA expression level in striatum in male and female groups (*n* = 3) were detected via quantitative PCR, including **(A)** Iba-1, **(B)** TNF-α, and **(C)** iNOS. Values are presented as mean ± SEM for three mice in each group. **(D,E)** CD11b protein level was detected via western blotting. *P < 0.05 and **P < 0.01 versus Vehicle groups, #*P* < 0.05 versus PJ34 + Vehicle groups. n.s., no significance.

To further confirm whether PARP-1 regulated microglial activation and aggravated post-stroke inflammation via Prx1, recombinant mouse Prx1 together with PJ34 were injected into the ischemic striatum, where microglia was abundantly clustered in both males and females. Quantitative PCR and western blotting were performed to evaluate the neuro-inflammation profiles. As shown in Figure 6, the exogenous recombinant Prx1 could block the inhibitory effects of PJ34 on post-stroke inflammation in the male MCAO mice,as the reduction of Iba-1, iNOS, and TNF-α mRNA levels induced by PARP-1 inhibition were reversed by Prx1. Consistently, reduction of mRNA level of Iba-1 and iNOS were also reversed by exogenous Prx1 in female MCAO mice, although TNF-α mRNA level was not significantly up-regulated (Figures 6A–C). Besides, Prx1 injection could also reverse the CD11b protein level reduction induced PJ34 in both males and females (Figures 6D,E). Collectively, these data showed that exogenous Prx1 rescued the inhibition of both microglial activation and neuro-inflammation induced by PARP-1 suppression.

## Discussion

This study has shown that delayed treatment of PARP-1 inhibitor at 48 h after ischemic stroke could prominently suppress microglial activation, and mitigate both post-stroke neuro-inflammation and neurological deficits. It is worthy to note that the regulatory effects of delayed PARP-1 inhibition on post-stroke inflammation are comparable in males and females. However, the extent of decline in inflammatory cytokines not completely consistent and improvement of neurological performance was more prominent in males. In addition, our study also demonstrated that Prx1 might be a critical mediator for the actions of PARP-1.

PARP-1, a major member of PARP family, plays an important role in a variety of biological functions, including DNA repair, genomic stability, and gene expression (Gibson et al., 2016). PARP-1 inhibitor has been approved for clinical treatments for several tumors, such as ovarian cancers (Lin and Kraus, 2017), microsatellite unstable cancers (Chan et al., 2019) and pancreatic cancers (Tuli et al., 2019), with satisfactory clinical effects and safety. Interestingly, the potential of PARP-1 inhibitor in non-tumor diseases has been put forward by some researchers and clinical physicians, especially in the scope of CNS diseases. In the brain, mild DNA damage activates PARP-1 to help DNA repair and cell survival. However, excessive PARP-1 activation results in an energy-consuming cellular process, which ultimately leads to neuronal cell death (Schreiber et al., 2006). In addition, PARP-1 has also been shown to modulate neuro-inflammation and adaptive immunity (Rosado et al., 2013). In the ischemic stroke, Koh et al. reported that PARP-1 inhibitor (3-AB, given at 15 min before MCAO) could reduce ischemic cell death and neuro-inflammatory response,with inflammatory cytokines including CD11b and IL-1β down-regulated in the 3-AB treatment group (Koh et al., 2004).Xu et al. (2016) found that PARP-1 inhibitor (PJ34, given immediately after MCAO and followed by one injection per day until brain harvest at 72 h after injury) could block post-stroke microglial activation and reduce MMP9 level. Overall, previous studies mainly focused on the effects of immediate PARP-1 inhibitor administration on post- stroke inflammation. However, few attention had been paid on whether delayed PARP-1 inhibitor treatment, especially after the acute phase of cell death, could also alleviate neuro-inflammation, which is of great significance. In this study, PARP-1 inhibitor PJ34 was given to male and female MCAO mice at 48 h after MCAO, and our results demonstrated that delayed delivery of PJ34 could significantly block post-stroke microglial activation, alleviate neuro-inflammation in male and female MCAO mice(Figures 1–3). *In vitro*, PJ34 was also found to inhibit LPS-induced primary microglial activation(Figure 4). Therefore, our findings indicated that delayed PARP-1 inhibition at 48 h could significantly alleviate neuro-inflammation, with a widen time window providing possibility of intervention.

It is widely acknowledged that the pathophysiology and functional outcomes of ischemic stroke are sexually dimorphic and age-dependent (Lang and McCullough, 2008; Shen et al., 2019). Tissue plasminogen activator (tPA) improves stroke outcomes for both sexes while females display more robust improvement in stroke outcome (Sohrabji et al., 2017). Despite greater age and higher rate of atrial fibrillation, females displayed comparable functional outcomes and greater years of optimal life after endovascular stroke thrombectomy compared with males (Sheth et al., 2019). PARP-1 was definitely over-activated in both males and females, and inhibition of PARP-1 was proven to dramatically reduce ischemia-induced PAR formation in both sexes (Yuan et al., 2009; Jog and Caricchio, 2013). Therefore, PARP-1 inhibitors hold promise in clinical treatment for ischemic stroke. However, it is controversial that whether gender dimorphism exists in the neuroprotective effects of PARP-1 inhibitor in stroke. Interfering PARP-1 signaling was reported to alleviate ischemic brain injury in males while aggravate brain injury in females (Yuan et al., 2009; Liu et al., 2011). In contrast, it was observed that PARP inhibition modulated microglial phenotypes, improved behavioral functions, and myelination during adulthood only in female mice following neonatal ischemia (Charriaut-Marlangue et al., 2018). On the contrary, the sex differences were not observed in some studies. In a primate study of stroke, the PARP inhibitor MP-124 showed comparable protective effects in both sexes (Matsuura et al., 2011). Likely, Du et al. (2004) showed that PARP inhibition was equally protective in sex-segregated primary cortical neurons exposed to nitrosative stress. In this study, we compared the innate gender-based proclivity in response to delayed PARP-1 inhibition in the experimental stroke. Our findings demonstrated that neuro-inflammation at 72 h after MCAO were comparably mitigated by delayed treatment of PARP-1 inhibitor PJ34 (Figures 1, 3), and PARP-1 inhibition was also found to remarkably alleviate LPS-induced sex-segregated primary microglial activation *in vitro* (Figure 4). Our microarray assay results indicated that delayed PARP-1 inhibition could differently affect inflammatory pathways in male and female MCAO mice. Besides, inflammatory cytokines reduction induced by delayed PARP-1 inhibition were not completely consistent in males and females. For instance, more significant reduction of iNOS and MMP9 by PARP-1 inhibition were found in male MCAO mice (Figures 1A,C). It was widely accepted that iNOS and MMP9 play critical roles in damaging blood–brain barrier integrity and aggravating ischemic brain injury (Ransohoff, 2016; Li et al., 2018). These differences might to some extent account for the more significantly improved neurological performance in males compared to females in stroke (Figures 1E,F), however, the detailed mechanism of which still need our further investigation.

Much effort has been paid to explore the detailed mechanisms underlying the actions of PARP-1 in modulating cell apoptosis, necrosis, and inflammation after ischemic stroke. PARP-1 has been proven to act as a co-activator in the NF-κB-mediated transcription, which was a key modulator regulating the expression of several elements of inflammation such as cytokines, chemokines, adhesion molecules, and inflammatory mediator (Rosado et al., 2013). In this study, substantial decreases of iNOS and MMP9 by PARP-1 inhibition were only found in male MCAO mice (Figures 1A,C). However, our findings in parallel demonstrated that delayed PARP-1 inhibition mitigated inflammatory molecules including Iba-1, CD11b, and IL-1 β (Figures 1A–D) in MCAO mice of both genders. To screen, we conducted a protein mass spectrometry to explore potential molecular mechanisms underlying the effects of delayed PARP-1 inhibition on the post-stroke inflammation. Prxs are a family of antioxidant enzymes that catalyze the reduction of peroxides (Bell and Hardingham, 2011), and Prxs were markedly increased during ischemic stress (Nakamura and Shichita, 2019). PRX1 is abundant in microglia, PRX2 to 5 in neurons, and PRX6 in astrocytes (Nakamura and Shichita, 2019). Recent evidence proposed that extracellular Prxs could be a damage-associated molecular pattern molecules (DAMPs) to activate the Toll-like receptor (TLR) 2 and TLR4, resulting in aggravated post-ischemic inflammation (Shichita et al., 2012; Nakamura and Shichita, 2019). Based on the findings of proteomic analysis, we further demonstrated that Prx1 were significantly down-regulated after PJ34 treatment in both males and females with Prx6 not affected, using western blotting assay (Figure 5). Consistently, the microglial activation and inflammatory response were prominently suppressed (Figures 3, 4). In addition, previous studies indicated that Prx1 could significantly induce the expression of TNF-α, IL-1β, and IL-6 via the Toll-like-receptor-4a-mediated NF-κB signaling pathway in stroke (Liu et al., 2016; Liu and Zhang, 2019). Prx1 level was also reported to be a biological marker for determining cerebral infarction onset (Richard et al., 2016). In this study, it was shown that exogenous recombinant Prx1 injection reversed the anti- inflammatory effects of PARP-1 inhibition (Figure 6), which further validated our hypothesis that PARP-1 inhibition might block microglial activation and alleviate neuro-inflammation via down-regulating Prx1.

## Conclusion

In conclusion, we for the first time investigated the effects of delayed PARP-1 inhibition on post-stroke inflammation in males versus females. Our findings revealed that delayed treatment of PARP-1 inhibitor could comparably suppress microglial activation and alleviate post-ischemic neuro-inflammation. We further proposed that Prx1 might be a critical factor involved in the actions of PARP-1 inhibitor in both males and females in the ischemic stroke. However, more significant reduction of iNOS and MMP9 induced by PARP-1 inhibition and better neurological functions were found in male MCAO mice, indicating gender differences of PARP-1 inhibitor treatment for stroke. Further investigations are needed to identify the detailed mechanisms of the sex-specific effects of PARP-1 inhibition in stroke.

## Data Availability Statement

The datasets generated for this study are available on request to the corresponding author.

## Ethics Statement

All experiments involving animals were approved by the Animal care and Use Committee at Nanjing University.

## Author Contributions

YX and YC conceived and designed the study. JC, XL, SX, and ZW performed the experiments and analyzed the data. YC, JC, MZ, and XZ wrote, revised, and checked the data analysis. All authors revised and approved the final version of the manuscript.

## Conflict of Interest

The authors declare that the research was conducted in the absence of any commercial or financial relationships that could be construed as a potential conflict of interest.
