# Supplementary material for: Delayed PARP-1 Inhibition Alleviates Post-stroke Inflammation in Male Versus Female Mice: Differences and Similarities
Source: Front Cell Neurosci. 2020 Apr 3;14:77. doi: 10.3389/fncel.2020.00077 (PMC7146057; doi:10.3389/fncel.2020.00077)
Supplement: Supplementary file 1 [file Data_Sheet_1.DOCX]

**Supplementary File**

**
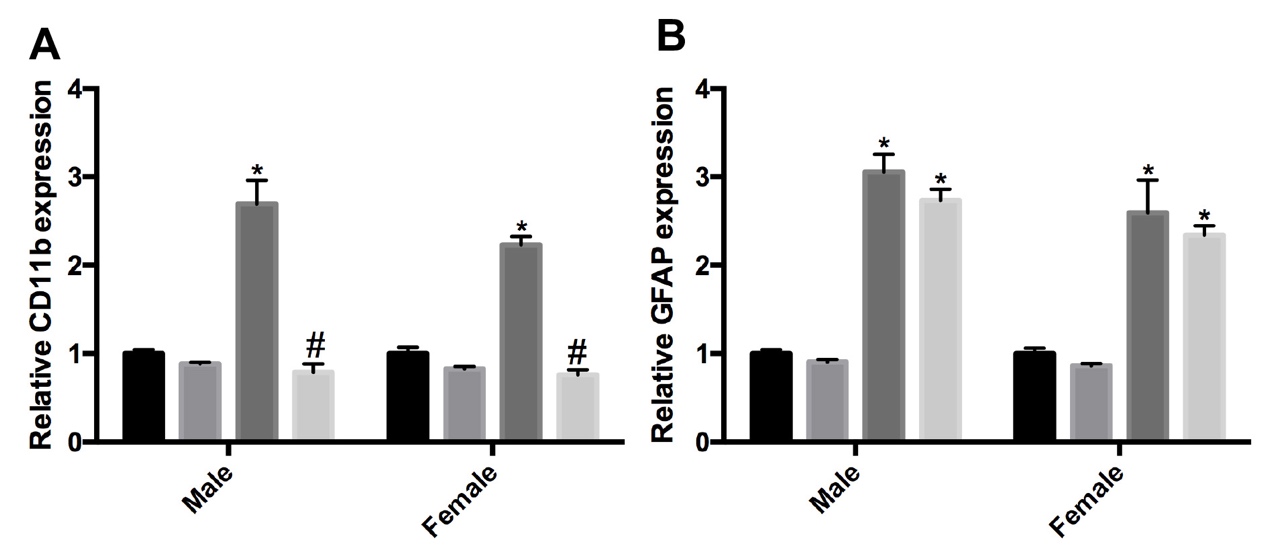
**

Supplementary Figure 1: (A) Quantitative data of blot band of CD11b in Figure 3. (B) Quantitative data of blot band of GFAP in Figure 3.
